# Precision spectroscopy of atomic helium

**DOI:** 10.1093/nsr/nwaa216

**Published:** 2020-08-31

**Authors:** Yu R Sun, Shui-Ming Hu

**Affiliations:** Hefei National Laboratory for Physical Sciences at Microscale, iChem Center, University of Science and Technology of China, Hefei 230026, China; CAS Center for Excellence in Quantum Information and Quantum Physics, University of Science and Technology of China, Hefei 230026, China; Hefei National Laboratory for Physical Sciences at Microscale, iChem Center, University of Science and Technology of China, Hefei 230026, China; CAS Center for Excellence in Quantum Information and Quantum Physics, University of Science and Technology of China, Hefei 230026, China

**Keywords:** precision spectroscopy, helium, quantum electrodynamics, fine-structure constant, nuclear charge radius

## Abstract

Helium is a prototype three-body system and has long been a model system for developing quantum mechanics theory and computational methods. The fine-structure splitting in the 2^3^P state of helium is considered to be the most suitable for determining the fine-structure constant α in atoms. After more than 50 years of efforts by many theorists and experimentalists, we are now working toward a determination of α with an accuracy of a few parts per billion, which can be compared to the results obtained by entirely different methods to verify the self-consistency of quantum electrodynamics. Moreover, the precision spectroscopy of helium allows determination of the nuclear charge radius, and it is expected to help resolve the ‘proton radius puzzle’. In this review, we introduce the latest developments in the precision spectroscopy of the helium atom, especially the discrepancies among theoretical and experimental results, and give an outlook on future progress.

## INTRODUCTION

Two electrons are much more than one. Helium is the simplest multielectron atom among a few ‘calculable’ systems. Its electronic energies cannot be solved analytically as the hydrogen atom, but can be numerically calculated based on quantum electrodynamics (QED) theory without using any adjustable parameters. Therefore, the helium atom has been an excellent platform for testing theories and has played a significant role in the development of the calculation methodology of multielectron systems. The full-quantum calculation of the helium atom has been achieved primarily through the variational method using the Hylleraas basis [1]. With succeeding progress achieved by theorists over the past several decades, it is now possible to calculate the nonrelativistic energies of the helium atom to arbitrary precision (see [2–7] and the references therein). By adding high-order QED and relativistic corrections, transition frequencies of the helium atom can now be calculated with over 10 digits [8]. At such an accuracy, the comparison with the experimental results becomes a stringent test of bound-state QED.

The 2^3^P state of ^4^He splits into three sublevels with intervals of 29 GHz and 2.3 GHz, respectively. In 1964, Schwartz [9] proposed that the 29 GHz interval is very suitable to determine the fine-structure constant α, which is the most important dimensionless constant in QED. Before that, the best value of α was derived from the 2P_1/2_-2P_3/2_ fine-structure splitting of hydrogen. However, it is challenging to improve accuracy due to the short lifetime of the 2*p* state of hydrogen. The lifetime of the 2^3^P state of helium is about two orders of magnitude longer, making it more promising to determine α in helium. In the last few decades, Drake, Pachucki and their colleagues have considerably improved the precision of the calculated intervals [10–15], which currently reaches 1.7 kHz, corresponding to a determination of α with an accuracy of 27 ppb. A comparison of α values derived from a variety of methods [16], from the anomalous magnetic moment of electron (*g*_*e*_ − 2) to the quantum Hall effect, presents a consistency check of QED in different fields of physics.

Since the atomic nucleus has a spatial distribution rather than a point charge, it induces shifts in the electronic energies of atoms. The energy shift from the calculated value based on a pointlike nucleus could be used to determine the nuclear charge radius. Since the terms unrelated to the nuclear mass cancel in the frequency difference between corresponding transitions of different isotopes, the isotopic shift can be calculated with better precision and used to determine the difference between two nuclear charge radii [17,18]. Different transitions involving the *s* states have been used for such measurements of different isotopes of helium, ^3^He, ^4^He, and even the short-lived isotopes ^6^He [19] and ^8^He [20,21]. The comparison can also be applied between normal electronic He (*e*-He) and the exotic μ-He where a muon particle replaces the electron. As an analogous comparison between *e*-H and μ-H, it would help to resolve the ‘proton radius puzzle’, which has received significant interest [22–26]. Moreover, it provides a test of the universality in the electromagnetic interactions of leptons.

Precision measurements of the helium atom also have other applications in testing fundamental physics and quantum chemistry calculations, as well as in metrology. Below we review the precision measurement studies of the helium atom from the above aspects and also prospect possible progress in this field.

## ENERGY LEVELS AND TRANSITIONS OF HE

**Figure 1.**
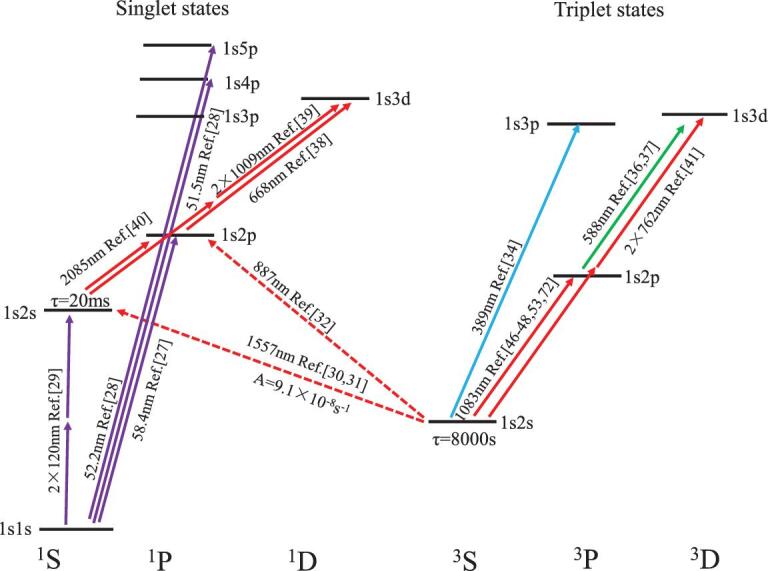
Energy levels and transitions of He.

The energy levels of the helium atom can be divided into singlet and triplet states according to the quantum number of the spin, as shown in Fig. 1. Transitions from singlets to triplets are spin forbidden. Measurements of the transitions from the ^1^S_0_ ground state of the helium atom are hindered by the difficulty in a vacuum ultraviolet light source. In 1996, Eikema *et al.* [27] recorded the 1^1^S-2^1^P transition at 58.4 nm. They realized a light source tunable at 58 nm using the fifth-harmonic generation of a pulsed laser. Later, Eikema *et al.* also measured the transitions from the ground state to the 4^1^P (52.2 nm) and 5^1^P (51.5 nm) states  [28]. The ultraviolet, optical frequency comb technique was applied in the frequency metrology, and a fractional accuracy of 1 × 10^−9^ was achieved in these measurements. The two-photon transition from the ground state to the 2^1^S state was also reported in the 1990s [29]. It is worth noting that there is a significant deviation of 180 (36)_exp_(48)_calc_ MHz between the result of the two-photon transition and the recently obtained theoretical calculation [8].

The helium atom has two long-lived metastable states, 2^1^S_0_ (τ ≈ 20 ms) and 2^3^S_1_ (τ ≈ 7800 s), and the latter is known as the metastable state with the longest lifetime in atoms. Theorists concluded that transitions from these two long-lived metastable states are sensitive to the QED effect, which can be verified by measuring the Lamb shift. Many of these transitions are in the near-infrared or visible range, where the best commercial lasers are available, being a significant advantage for precision measurements. Metastable helium atoms have a very high internal energy of about 20 eV. Therefore, they can be directly detected with a quantum efficiency of almost 100%. The very weak 2^3^S-2^1^S transition has a line width of 8 Hz, which has been considered as a potential atomic clock transition. Precision spectroscopy measurements on the triplet to singlet transitions, including 2^3^S-2^1^S [30,31] and 2^3^S-2^1^P [32], have been carried out by a group in Amsterdam led by Wim Vassen. They prepared cold helium atoms in the 2^3^S metastable state using a dipole trap before the spectroscopy measurement of the forbidden transitions. An alternative method is to measure them in an atomic beam. It was proposed by van Leeuwen and Vassen [33], but no experimental measurement has so far been reported.

The 2^3^S_1_-2^3^P_*J*_ transition is particularly interesting to determine the nuclear charge radius, as well as the fine-structure constant from the intervals among the 2^3^P_*J*_ (*J* = 0, 1, 2) levels, making it the focus of extensive study in the last twenty years, which will be discussed in more detail in the next two sections.

The fine-structure splitting in the 3^3^P_*J*_ (1*s*3*p*) state of the helium atom has also been studied by Mueller *et al.* [34] through the measurement of the 2^3^S-3^3^P transition at 389 nm. The experimental accuracy was improved to 30 kHz, agreeing well with the calculated results. Drake and his colleagues also calculated the ionization energy of the 3D state and reached a very high accuracy of 20 kHz [35]. For this reason, this value has been used as a reference of the ionization energies of other energy levels of helium. Many recent measurements related to the 3D state of helium were accomplished by Libang Wang and his colleagues, including the 2^3^P-3^3^D transition at 588 nm [36,37], the 2^1^P-3^1^D_2_ transition at 668 nm [38], the two-photon transition of 2^1^S-3^1^D_2_ [39] and the 2^1^S-2^1^P transition at 2058 nm [40]. The 2^3^S-3^3^D interval has been measured by Dorrer *et al.* [41] using two-photon spectroscopy in 1997, and it agrees with the result from the measurement of the 2^3^S-2^3^P_1_ and 2^3^P_1_-3^3^D_1_ transitions. However, there are discrepancies between the experimental and theoretical results on the transitions involving the 3D state: 1.6(1.3) MHz for 2^3^S_1_-3^3^D_1_, 1.4(0.7) MHz for 2^3^P_0_-3^3^D_1_ and 1.7(0.5) MHz for 2^1^P_1_-3^1^D_2_. The latest calculation of the 3D state [42] corrected the ionization energy by 0.3 MHz, 10 times the previously stated uncertainty, but could not explain the deviations. This indicates that more independent theoretical and experimental investigations on this state are needed.

## THE 2^3^P_*J*_ SPLITTING AND THE FINE-STRUCTURE CONSTANT

The electronic energies of helium can be expanded in a power series of the fine-structure constant α by adding high-order QED corrections:

(1) \begin{eqnarray*} E (m, \alpha ) & =& m \alpha ^2 \mathcal {E}^{(2)}+ m \alpha ^4 \mathcal {E}^{(4)} + m \alpha ^5 \mathcal {E}^{(5)} \nonumber\\ +\, m\alpha ^6 \mathcal {E}^{(6)} + m\alpha ^7 \mathcal {E}^{(7)}+\cdots . \end{eqnarray*}

Here *m* is the (reduced) mass of the electron. The fine-structure splitting is determined by the second term, which is proportional to *m*α^4^ (equivalent to *R*_∞_α^2^, where *R*_∞_ is the Rydberg constant). The 32 GHz interval 2^3^P_0_-2^3^P_2_ is larger than any other fine-structure splitting in helium or hydrogen atoms, and also has a relatively long lifetime of 98 ns. Therefore, it is best for determining the fine-structure constant α in atoms [9]. Recent reports indicate that the 2^3^P fine-structure splitting can also be used to search the anomalous spin interaction [43].

**Figure 2.**
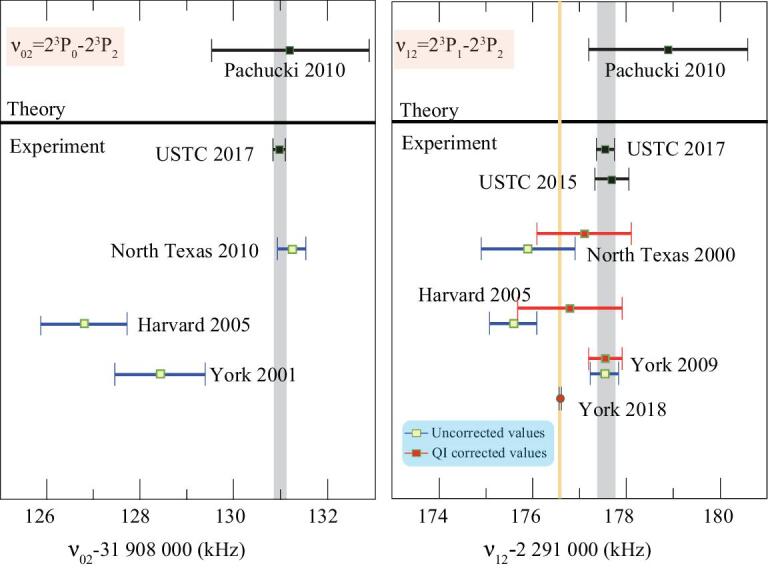
The calculated and experimental intervals in the 2^3^P_*J*_ fine structure of ^4^He. Red points indicate the corrections due to the quantum interference (QI) effect. Note that the quantum interference effect is negligible for the 32 GHz splitting 2^3^P_0_-2^3^P_2_.

During the last twenty years, several groups worldwide have carried out measurements of fine-structure splittings to subkilohertz accuracy using different experimental methods. The theoretical development in the calculation of the 2^3^P_*J*_ fine structure was respectively carried out by Drake from Windsor University and Pachucki from Warsaw University and their coworkers. An accuracy of less than 0.2 kHz was claimed in calculations [12,13] to the order of *m*α^7^. However, comparison of the experimental and calculated results shows a significant discrepancy of 10 times the stated deviation. The reason was unknown at that time. Theorists believed that it could be a result of the contribution from the higher-order QED corrections of *m*α^8^ [13]. This deviation has aroused great concern in this field, which also triggered our experimental measurements in an atomic beam [44]. In 2009, Pachucki and Yerokhin re-examined the *m*α^7^ term in the calculation, and corrected previous evaluation of the relativistic Bethe logarithms [14]. The new theoretical result [15] agreed with the experimental results (see Fig. 2), and the consistency among different calculations was also verified [45]. This presents an excellent example of how the experimental measurements test theoretical calculations and promote the development of theories. There are still significant difficulties in the calculation of the *m*α^8^ term, but Pachucki and Yerokhin [15] gave an estimation of about 1.7 kHz for the size of the *m*α^8^ term. This term is the present accuracy limit of the calculated fine-structure splitting of the 2^3^P manifold, which limits the determination of α with an accuracy of 27 ppb. However, the *m*α^8^ term could be identified from the measurements of other He-like ions, which would help to improve the theoretical precision of helium [8].

Several different methods have been applied in precision spectroscopy of the 2^3^P_*J*_ fine structure of atomic helium. The representative ones are: the saturated absorption spectroscopy in a gas cell by Zelevinsky *et al.* [46] from Harvard University, microwave spectrum measurement by Borbely *et al.* [47] from York University and laser spectroscopy in an atomic beam by Smiciklas and Shiner [48] from the University of Texas. By 2010, the theoretical and experimental results are consistent. However, when the experimental accuracy was improved, discrepancies among the results from different measurements turned out to be significant compared to the stated uncertainties. Hessels [49–51] proposed a frequency shift arising from the quantum interference effect due to adjacent states, which was not considered in previous studies. In particular, the shift in the laser spectroscopy measurement of the small fine-structure split of 2.3 GHz can reach a few kilohertz to tens of kilohertz, which considerably exceeds previously claimed uncertainties.

**Figure 3.**
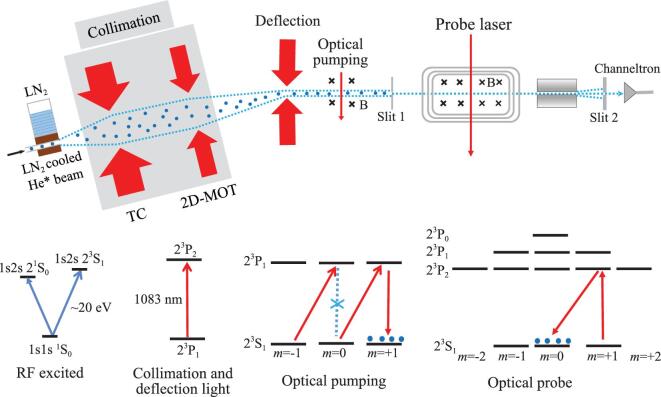
The Hefei experimental setup built for laser spectroscopy of helium. Metastable helium atoms were produced in a tube cooled by liquid nitrogen. The atomic beam was then collimated by transverse cooling (TC) and a two-dimensional magneto-optical trap (2D-MOT), and then deflected from the original beam, all by lasers on resonant with the 1083 nm 2^3^S-2^3^P transition. Atoms at the 2^3^S_0_ state were transferred to *m* = ±1 levels by optical pumping, and deflected by a Stern–Gerlach magnetic field. When the probe laser scanned over resonance of the 2^3^S-2^3^P transition, some atoms were excited and transferred again to the *m* = 0 level by spontaneous decay, which could pass through the Stern–Gerlach magnetic field and reach the detector.

**Figure 4.**
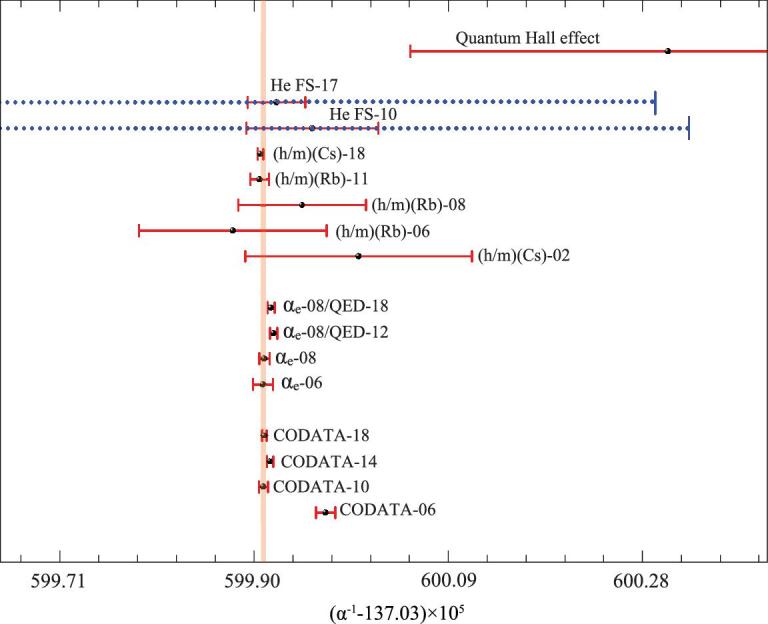
Values of the fine-structure constant α derived from different methods: anomalous magnetic moment of electron (*g*_*e*_ − 2) [54,55] (and those after theoretical revision [56,57]), atomic recoil (*h*/*M*) [58–62], fine structure of helium [53,64] and the quantum Hall effect [63]. The CODATA recommended values [16,63,65] and the latest CODATA-18 value from the National Institute of Standards and Technology (NIST) are given at the bottom of the figure. The theoretical and experimental error bars of the α value derived from the helium 2^3^P_*J*_ fine-structure splitting are given by blue dashed and solid red lines, respectively.

We have developed an apparatus for laser spectroscopy of helium in a collimated atomic beam. A schematic configuration of the setup is shown in Fig. 3. We used radio-frequency discharge to produce metastable helium atoms and laser cooling techniques to prepare a collimated bright atomic beam of metastable helium at the 2^3^S state. Optical pumping was used to evacuate atoms at the *m* = 0 level, and a weak spectroscopy laser repopulated the *m* = 0 level. Only atoms at the *m* = 0 level can pass through a Stern–Gerlach magnetic field where the magnetic field gradient was about 0.5 T/cm, and finally hit the electron multiplier detector. The spectroscopy probing zone was shielded by a μ-metal, and the probe laser frequency was referenced to a stable laser and eventually to a frequency comb. Using this apparatus, we first measured the 2^3^P_1_-2^3^P_2_ interval of 2.3 GHz [52]. We obtained an accuracy of 0.3 kHz in 2015 and then improved this to 0.19 kHz in 2017 [53]. After taking into account the quantum interference correction of about 1.2 kHz, both our results agreed with those obtained from very different experimental methods. This result experimentally validated the effects of quantum interference and explained the reasons for the discrepancies among different experimental groups over the years. We also determined the large split in the 2^3^P_0_-2^3^P_2_ fine structure, which is 31 908 130.98(13) kHz, being the one with the highest precision. It deviates from the theoretical value [15] by only 0.22 ± 0.13_exp_ ± 1.7_theo_ kHz. The comparison of the experimental and theoretical results presents a verification of the *m*α^7^ term QED corrections for the first time. If the theoretical calculations reach the same accuracy as our experimental result, it will give an independent determination of the fine-structure constant at an accuracy of 2 ppb [53]. The value can be compared with α values determined from very different methods: from the anomalous magnetic moment of electron (*g*_*e*_ − 2) [54–57]), from the atomic recoil measurements that determine the *h*/*M* values of Cs [58,59] and Rb [60–62] and from the quantum Hall effect [63]. These values are illustrated in Fig. 4. Such a comparison presents a verification of the consistency of QED in different fields of physics.

Recently, Hessels’ group at York University obtained a breakthrough in the microwave spectroscopy of the 2.3 GHz splitting. The uncertainty was dramatically reduced to 25 Hz by using the frequency-offset separated oscillatory fields method [66]. However, as shown in Fig. 2, the result deviates by 940 Hz from the group’s previous 2009 result [47], by 2.9σ from our 2015 result [52] and by 4.9σ from our 2017 result [53]. Considering that our 2017 result agrees very well with our 2015 result, and our setup has been rebuilt between 2015 and 2017, we can eliminate the deviation due to the repeatability of our measurements. This indicates that there is an unknown systematic error in the experimental methods, and more independent measurements are needed. Hessels and his colleagues are also going to conduct the measurement of the large splitting of 2^3^P_0_-2^3^P_2_, and we are on the way to upgrading the experimental setup and remeasuring the fine structure, which may help to reveal the reason for the deviation.

## NUCLEAR CHARGE RADIUS FROM SPECTROSCOPY

Since the *s* electron has a certain probability penetrating the nucleus, the charge distribution of the nucleus has an influence on the energies of electrons in *s* orbitals. If the point charge model is used to calculate the atomic transition frequency, the atomic nuclear charge radius can be estimated from the frequency difference to the experimental value. Because it is challenging to measure transitions from the ground state (1^1^S) of helium, transitions involving the 2^1^S/2^3^S states are most suitable for determining the atomic nucleus charge radius. Pachucki and his colleagues calculated the 2S-2P transition frequencies with an accuracy of 2 MHz, and predicted that the calculation accuracy can be improved to 10 kHz in the near future [8]. Such accuracy is expected to yield a determination of the helium nuclear charge radius with a fractional accuracy of one thousandth, which has a subsequent impact in fundamental physics.

Similar to the study on the exotic atom of μ-H, where an electron is replaced by a muon, spectroscopy on μ-He is being carried out by a group led by Pohl [67,68]. QED calculation of the energy levels of μ-He has also been conducted [69,70]. A comparison of the theoretical and experimental results of μ-He provides a determination of the charge radius of the helium nucleus, which can be compared with that obtained from the *e*-He studies. Under the framework of the standard model, the charge radii obtained from μ-He and *e*-He should be the same. If there is an abnormality in the comparison, it may be an indication of new physics. Analogous to the studies of the proton size based on *e*-H and μ-H, the study of the charge radii of helium nuclei is expected to play an important role in testing the standard model, which also provides another perspective on the ‘proton radius puzzle’.

**Figure 5.**
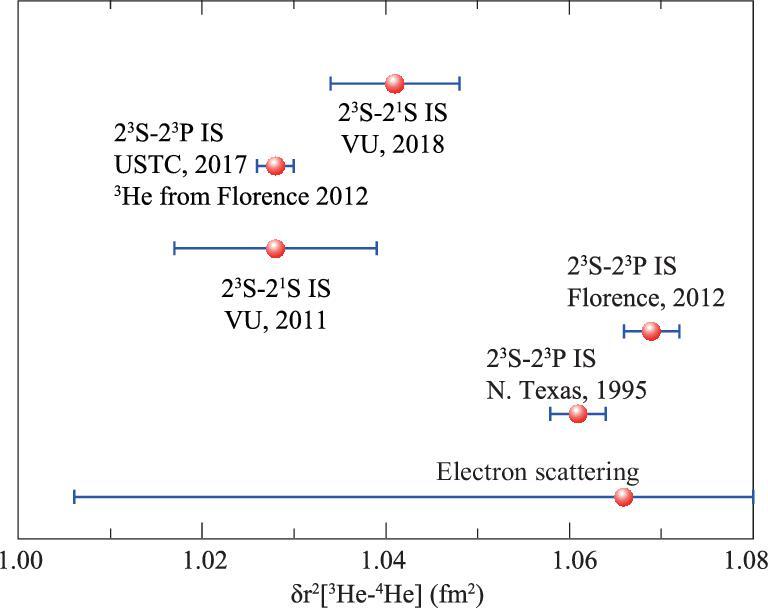
Differences between the squares of nuclear charge radii of ^4^He and ^3^He, δ*r*^2^ = *r*^2^(^3^He) − *r*^2^(^4^He), obtained from different sources: from the 2^3^S-2^3^P transition [71,73,76], from the 2^3^S-2^1^S transition [30,31] and from electron scattering [77].

Moreover, the measurement of the isotopic shift between ^3^He and ^4^He will give a determination of the difference between these two nuclei, δ*r*^2^ = *r*^2^(^3^He) − *r*^2^(^4^He). Since some common terms cancel in the isotopic shift, the calculation accuracy can be improved by another factor of 10 [8], which also makes it very suitable for testing QED. Two transitions have been applied to determine δ*r*^2^, one is 2^3^S-2^3^P and the other is 2^3^S-2^1^S. The two methods have little difference in theoretical calculations, and the nuclear charge radius differences obtained from both methods should agree with each other. However, they have a standard deviation of 4σ, as shown in Fig. 5. It may be either a result of unknown systematic error in the measurements or a potential indicator of new physics. Since the measurement of μ-^3^He is also feasible, the study could be further enriched. Therefore, related research has received widespread interest in the field of precision measurement physics.

Shiner and his colleagues measured the 2^3^S_1_-2^3^P_0_ transition of ^3^He and ^4^He and derived the isotopic shift in the 1990s [71]. Later the application of frequency comb in metrology allowed for precise determination of the absolute laser frequency. The group at Florence measured the 2^3^S-2^3^P transition of helium in an atomic beam using saturation spectroscopy [72] and reported central frequencies for ^4^He and ^3^He [73] as 276 736 495 649.5(2.1) kHz and 276 702 827 204.8(2.4) kHz, respectively. Using this isotopic shift, they derived a δ*r*^2^ value of 1.069(3) fm^2^ [8], which is shown in Fig. 5.

In 2017, we measured the 2^3^S-2^3^P transition of ^4^He using the experimental setup shown in Fig. 3. Surprisingly, the frequency obtained from our measurement deviates from the Florence group by 50 kHz, 20 times the standard deviation. If we compare the experimental methods applied in both studies, we can see that the saturation spectroscopy method used by the Florence group has an advantage in eliminating the Doppler effect. However, relatively high laser power applied in their measurements subsequently induced a large power-related recoil induced shift. The shift was as large as 90 kHz, and the evaluation was rather complicated. It should be noted that the quantum interference correction was not considered in that study. According to the estimation by Marsman *et al.* [51], under the experimental conditions of the Florence group, the influence on the saturation spectroscopy measurement may exceed 10 kHz. In our experimental measurements, the power of the probing laser was relatively low, and our analysis [74] shows that the power-induced frequency shift in our measurement was negligible. The contribution from the quantum interference effect could also be assessed more accurately in our measurements. However, our present experimental accuracy is mainly limited by the Doppler shift (approximately equal to 1 kHz). We are now upgrading our experimental setup by using a Zeeman slower to reduce the longitudinal velocity of the helium atoms in the atomic beam from 1000 m s^−1^ to about 100 m s^−1^, which will considerably reduce the first-order Doppler shift down to about 100 Hz. Moreover, it will allow an accurate assessment of the Doppler effect by comparing the results obtained at different velocities.

The 2^3^S-2^1^S transition at 1557 nm of helium is doubly forbidden, which has a very low transition rate of 9.1 × 10^−8^ s^−1^ and a very narrow natural line width of 8 Hz. In 2011, Vassen and his colleagues first measured this transition using cold atoms in a dipole trap [30]. They observed a line width of 90 kHz and determined the frequencies of ^4^He and ^3^He transitions with uncertainties of 1.8 kHz and 1.5 kHz, respectively. The result of the ^3^He-^4^He nuclear charge radius difference δ*r*^2^ derived from their 2^3^S-2^1^S measurements is shown in Fig. 5. Note that δ*r*^2^ is less sensitive to the 2^3^S-2^1^S transition than the 2^3^S-2^3^P transition. Recently, Vassen’s group implemented [31] the dipole trap of ^4^He at a magic wavelength of 319.8 nm, and reduced the uncertainty in the 2^3^S-2^1^S transition frequency of ^4^He to 0.2 kHz. However, a discrepancy of 4σ in δ*r*^2^ remains between the new result and that of the Florence group.

Interestingly, if we combine our 2^3^S-2^3^P transition ^4^He result with that of ^3^He obtained by the Florence group (which is the only available one at this time), we can derive a ^3^He-^4^He nuclear charge radius difference, which is also shown in Fig. 5. It is close to that derived from the 2^3^S-2^1^S transition by Vassen’s group. We cannot say that this discrepancy is solved in this way, but it indicates that the problem would be more likely in the experimental measurements, and it is necessary to conduct an independent measurement of the 2^3^S-2^3^P transition of ^3^He. Note that the quantum interference effect in the laser spectroscopy of ^3^He could be stronger due to the presence of a hyperfine structure. Since the hyperfine intervals have been calculated precisely by Pachucki *et al.* [75], the center frequency of the 2S-2P transition of ^3^He can be derived from the transition frequency to a single level in the 2^3^P state together with the calculated hyperfine splittings. The difficulty of complicated quantum interference effects could be circumvented by measuring the transition to the most isolated level (*F* = 1/2, *J* = 0) in the 2^3^P state of ^3^He. It is also worth noting that the calculated value given by Pachucki *et al.* [8] is only 0.2 MHz lower than the experimental value, which has an uncertainty of 2 MHz due to the yet unknown *m*α^7^ QED corrections. An improvement of the theoretical calculation that takes into account the *m*α^7^ term could potentially reduce the uncertainty to about 10 kHz. With that, the comparison of the experimental and theoretical frequencies of the 2^3^S-2^3^P transition will give a determination of the nuclear size with an uncertainty of 0.1%.

## OTHERS

Thanks to the simplicity of the helium atom and the development of theoretical methods, the calculation accuracy has been continuously improved over the past few decades. The energy levels and wave functions of the helium atom have been calculated with very high precision, which also results in very accurate calculations of other properties of helium.

The 2^3^S and 2^1^S metastable states of helium have very high internal energies, and the potential energy curve between metastable helium atoms can be calculated very accurately. Therefore, by studying the collisions among cold helium atoms, we can study the elastic collision [78] and the collision-induced ionization [79,80], and also test the computational methods [81,82].

Being only heavier than the hydrogen atom, helium is also very attractive for atom interferometry studies. The helium atom interferometer [83] was among the first experiments in atom interferometry, and the study is still evolving, playing an essential role in testing basic quantum mechanics [84,85]. The use of helium atomic interference will also allow independent high-precision measurement of the fine-structure constant through the recoil velocity measurement (*h*/*M*) [86].

The dynamic polarization of the helium atom can also be calculated accurately. Because the lowest excited state contributes negative dynamic polarizability, which could be canceled by the contributions from other excited states, there could be some wavelengths such that the atomic polarizability vanishes to zero. These wavelengths are called the tune-out (or magic-zero) wavelengths. A comparison of the experimental and theoretical tune-out wavelengths provides a nonenergy test of QED. Recently, Henson *et al.* [87] measured the 413 nm tune-out wavelength of metastable (2^3^S_1_) helium at an accuracy of a few parts per million, which agrees well with the theoretical result.

The atomic polarizability can be used to calculate the gas refractive index, which can eventually be converted to the gas density. Therefore, an optical measurement of the refractive index of the helium gas can be used to determine the gas density (pressure). This method is expected to become a primary standard for the gas density (pressure), replacing the current gas pressure standard based on mercury. Experimentally, it has been straightforward to determine the change in the resonance frequency of an optical cavity containing sample gases, which is proportional to the refractive index of the gas. The measurements now reach an accuracy at the parts per million level [88]. However, due to the penetration of the helium atom into the cavity material (usually made of ultra-low-expansion glass), it leads to a systematic error currently more significant than 20 ppm. The groups at NIST and Physikalisch-Technische Bundesanstalt are developing stable optical cavities made of other materials [89], which may lead to a new pressure standard in the future.

Precision spectroscopy of other heliumlike medium- to high-*Z* ions [90–93] can also provide tests of higher-order quantum electrodynamics corrections. Note that the accuracy of the calculated fine-structure splitting of helium is currently limited by the correction of the order of *m*α^8^, which appears to be too complicated to be accomplished in the near future [8]. However, the theoretical precision could be improved by identifying the *m*α^8^ contribution in a light heliumlike ion such as Li^+^ or Be^2+^ and rescaling it to helium. Therefore, the study of fine-structure splittings in these heliumlike ions could help to improve the accuracy of the determination of α from helium spectroscopy.

## CONCLUSION

As the simplest multielectron atom, precision spectroscopy of helium has achieved remarkable progress in the last century. Based on the nonrelativistic QED expansion method, QED corrections as high as the *m*α^7^ terms have been included in the calculation of the electronic energies of helium, toward an accuracy at the subparts-per-billion level. This makes the precision spectroscopy of helium an ideal platform for testing the QED theory and developing calculation methods for multielectron atoms. The progress of helium atom interferometry will create new possibilities in fundamental physics, such as a new determination of the fine-structure constant independent of QED calculations.

The fine structure of the 2^3^P state of helium can be used to determine the fine-structure constant α with an accuracy at the 10^−9^ level. However, there are still great challenges in both the experimental and theoretical aspects: there is a deviation of 4σ between the latest laser spectroscopy measurement and the most recent microwave measurement; the calculation has been limited by the unknown *m*α^8^ QED correction.

The 2^3^S-2^3^P and 2^3^S-2^1^S transitions have been used to determine the nuclear charge radius of the helium atom. In particular, the isotope shift between ^4^He and ^3^He can be used to determine the nuclear charge radii difference. However, there are significant deviations between the results obtained by different research groups, and new independent measurements are needed to clarify this discrepancy. At the same time, the comparison between the normal electronic helium and the exotic muonic helium can play an essential role in solving the proton radius puzzle and even in searching for new physics beyond the standard model.
